# The cognitive and language abilities after stimulation in children aged less than 3 years iodine deficiency area

**DOI:** 10.1186/1687-9856-2013-S1-P150

**Published:** 2013-10-03

**Authors:** Asri Purwanti, Fitri Hartanto, Inikke

**Affiliations:** 1 Department of Pediatrics, Faculty of Medicine, Diponegoro University/Dr. Kariadi Hospital Semarang

## 

Iodine Deficiency Disorders (IDD) become a health problem in Indonesia and other developing countries. The influences of IDD on children’s intellectual are irreversible, but on the other hand it is said that early stimulation affects brain development. The aims of this study was to determine the cognitive and language abilities after stimulation in children aged less than 3 years in IDD area.

This study was a randomized controlled trial. Children aged 1-3 years, born and live in Wonosobo, one of the IDD area in Central Java, Indonesia (IDD 2008 mapping included this area in moderate endemic area) were test by Caput Scale, Otoacoustic emission (OAE) and Urine Iodine Excretion (UIE), then they were stimulated and monitored during 3 months. The data was analyzed by Mann-Whitney analysis, statistically significant if *p*<0.05 (the confidence interval is 95%).

One hundred thirty children were enrolled in this study. The mean age of subjects were 23.24 ± 6.91 months. Median UEI value was 175 µg/L. In IDD area, the value before and after stimulated were as follows : the cognitive ability (79,9 vs 85,1), language skills/CLAMS (80 vs 86,2), visual motor/CAT(80,2 vs 86,1). In non IDD area, before and after stimulated ;cognitive ability (84,7 vs 93,5), CLAMS(82,3 vs 91,0), CAT(82,4 vs 91,2). There were increasing the level cognitive and language after stimulation in IDD area and non IDD area,6 and 9 point respectively.

Our result suggest that family stimulation plays a role in improving children’s cognitive and language abilities in both areas of iodine deficiency or not, but there is varience between two groups before and after stimulation, where cognitive and language abilities of children in non IDD area higher then children in IDD area. Therefore controlling IDD program still become a main problem beside stimulation.

